# Association between abdominal obesity and asthma: a meta-analysis

**DOI:** 10.1186/s13223-019-0333-6

**Published:** 2019-03-22

**Authors:** Di Jiang, Liwen Wang, Chenxiao Bai, Ou Chen

**Affiliations:** School of Nursing, Shandong University, 44 West Wenhua Road, Jinan, Shandong China

**Keywords:** Asthma, Abdominal obesity, Waist circumference, Meta-analysis

## Abstract

**Background:**

Studies evaluating the association between abdominal obesity and asthma yielded conflict results. Whether abdominal obesity is positively associated with asthma remains unclear.

**Objective:**

To quantitatively determine the association between abdominal obesity and asthma.

**Methods:**

Databases including PubMed, Web of Science, China National Knowledge Infrastructure, China Biology Medicine disc, Chinese Scientific and Technological Journal Database and Wanfang Data were searched up to February 2018 to collect all relevant studies. Reference lists of related articles were also checked. After study selection and data extraction, meta-analysis was conducted to calculate the pooled odds ratio (OR) and corresponding 95% confidence interval (CI). Subgroup analyses by study design and age groups of participants were further performed. Publication bias was assessed via Begg’s rank correlation and Egger’s linear regression methods.

**Results:**

A total of 13 studies were included in the final meta-analysis, including 2 case–control studies, 6 cohort studies, and 5 cross-sectional studies. Our meta-analysis observed a positive association between abdominal obesity and asthma (OR = 1.47, 95% CI 1.35–1.59). No evidence of heterogeneity (I^2^ = 10.7%) or publication bias (Begg’s test P = 0.200, Egger’s test P = 0.146) was found. Subgroup analyses by study design and age groups of participants obtained consistently positive results across subgroups. Moreover, our meta-analysis observed similar results when considering this association separately in males and females (Males: OR = 1.37, 95% CI 1.18–1.58; Females: OR = 1.39, 95% CI 1.22–1.58). In addition, the association between abdominal overweight and asthma was further explored in this meta-analysis and the pooled OR and 95% CI was 1.13 (1.03, 1.24), indicating that there is a dose–response relationship between abdominal weight status and asthma.

**Conclusions:**

Our meta-analysis shows a positive association between abdominal obesity and asthma. Moreover, this association is similar in males and females. In addition, our meta-analysis indicates that there is a dose–response relationship between abdominal weight status and asthma. Therefore, addressing abdominal obesity issue is of great importance. More studies are needed in the future to clarify the association between abdominal obesity and asthma.

## Background

As one of the most common chronic respiratory diseases, asthma affects about 358 million people worldwide [1]. It is projected that by 2025, this number will increase to 400 million [2]. As a major cause of disability, absenteeism, and huge medical expenses, asthma places a substantial burden on the whole society [3, 4]. The total annual cost of asthma, including the direct medical cost of asthma (such as hospitalization and pharmacological treatment) and the indirect nonmedical cost of asthma (such as missed workdays and school days), is estimated to be EUR 19.3 billion in Europe [5] and $81.9 billion in the United States [6]. Asthma has become a major public health concern worldwide.

Obesity, as is known to all, is also a worldwide public health problem. In 2015, approximately 107.7 million children and 603.7 million adults worldwide were obese, accounting for about 30% of the world’s population [7]. A recent study that analyzed data from 195 countries around the world found that the prevalence of obesity has doubled in more than 70 countries over the past 25 years [7]. The increasing prevalence of obesity in recent years has made obesity-related health risks a global concern. Obesity has been generally recognized as an important risk factor for many chronic diseases such as cardiovascular disease, type 2 diabetes and cancer [8, 9]. A total of 4 million deaths and 120 million disability-adjusted life-years worldwide were estimated to be related to excess body weight in 2015 [7].

A growing number of studies have showed a significant association between obesity and asthma [10–13]. Moreover, this association has been further confirmed in several meta-analyses [14–17]. However, the “obesity” discussed in these studies, strictly speaking, refers to “general obesity”, which is typically measured by body mass index (BMI) [10]. As a crude measure of obesity, BMI can not discriminate between muscle mass and body fat, nor can it reflect body fat distribution [18]. Recently, however, more and more attention has been paid to body fat distribution, more precisely, to abdominal obesity. Abdominal obesity is a condition of having excess fat in the abdomen. There is growing evidence that abdominal obesity may be a key contributor to obesity-related health risks [19, 20]. Measures of abdominal obesity, such as waist circumference (WC), waist to height ratio (WHtR), waist hip ratio (WHR), and conicity index (CI), have been found to be better predictors of some obesity-related diseases such as cardiovascular disease and type 2 diabetes, compared to BMI [20–22]. Whether abdominal obesity also plays a more important role in asthma is worth exploring.

In spite of this, studies on the association between abdominal obesity and asthma are lacking. The findings of the existing studies are inconsistent. A case–control study [23] demonstrated that a 10 cm increase in WC was related with a 40% increase in the odds of developing asthma. Another study [13] showed that even among normal weight women (measured by BMI), if they had a WC of more than 88 cm, the risk of asthma would still be higher (OR = 1.37, 95% CI 1.18–1.59). But a study from Brazil [24] did not find a significant association between abdominal obesity and asthma (OR = 0.93, 95% CI 0.74–1.16). Moreover, when this association is considered separately in males and females, the results of previous studies are also controversial. Some studies [10] observed that this association was similar in males and females, some [12, 25–27] found that this association existed only in females or was stronger in females than in males, while others [28, 29] obtained exactly the opposite results. Few meta-analyses have been conducted to explore the association between abdominal obesity and asthma.

Therefore, we conducted this meta-analysis to quantitatively determine the association between abdominal obesity and asthma. Different from previous meta-analyses published in this field [14–17], our meta-analysis focused on abdominal obesity (measured by WC, WHtR, WHR, or CI) rather than general obesity (measured by BMI). Moreover, sex-specific association was also explored in this meta-analysis.

## Methods

### Literature selection

Databases including PubMed, Web of Science, China National Knowledge Infrastructure, China Biology Medicine disc, Chinese Scientific and Technological Journal Database and Wanfang Data were searched up to February 2018 to collect all relevant studies by two researchers independently. The search strategy was as follows: (“abdominal obesity” OR “central obesity” OR “visceral obesity” OR “abdominal adiposity” OR “central adiposity” OR “visceral adiposity” OR “waist circumference” OR “waist size” OR “waist hip ratio” OR “waist to hip ratio” OR “waist to height ratio” OR “conicity index”) AND (“asthma” OR “bronchial asthma”). In addition, reference lists of related original and review articles were also checked to find additional studies that may meet the eligibility criteria.

### Eligibility criteria

Eligible studies had to meet the following criteria: (1) were original epidemiological studies (of any design); (2) related to the association between abdominal obesity and asthma; (3) clearly stated the definition and measurement of abdominal obesity; (4) reported odds ratios (OR) or risk ratios (RR) and their 95% confidence intervals (CI), or provided sufficient data from which these measures could be calculated; (5) published in English or Chinese. Studies were excluded if they: (1) were animal studies; (2) were editorials, comments, or literature reviews; (3) reported overlapped data; (4) contained incomplete data which was still unavailable after contact with the author.

The titles and abstracts of the retrieved studies were screened according to the above criteria by two researchers independently. The full texts of the potentially relevant studies were then obtained and strictly assessed. Disagreements were resolved by discussion with a third researcher.

### Data extraction and synthesis

Data extraction was conducted by two researchers separately. Discrepancies were resolved by discussion with a third researcher. The following data were extracted from each eligible study: first author, country, year of publication, year of study, study design, study population, sample size, definition of abdominal obesity, anthropometric measures of abdominal obesity, adjusted OR or RR and their corresponding 95% CI, and confounders that were controlled in the study. If no effect estimate was provided in a given study, OR or RR and 95% CI were calculated from the raw data presented in the study. The authors were contacted for further information when necessary.

### Quality assessment

For cohort studies and case–control studies, the quality were assessed by the Newcastle–Ottawa Scale (NOS). The NOS evaluates a study based on three major aspects: the selection of the study groups, the comparability of the groups, and the ascertainment of the exposure or outcome of interest [30]. For cross-sectional studies, the Agency for Healthcare Research and Quality (AHRQ) recommended criteria [31] was used to evaluate the quality. The criteria consists of 11 items, each of which has an answer of either “yes”, “no”, or “unclear”. Quality assessment was performed by two researchers independently. Disagreements were resolved by discussion with a third researcher.

### Statistical analysis

Where the identification of abdominal obesity adopted consistent anthropometric measures across studies and the final results were presented in a similar fashion, meta-analysis was performed to calculate the pooled OR and associated 95% CI. Moreover, sex-specific association was further explored in this meta-analysis. When a study reported data for males and females separately, a fixed effect model was used to calculate the pooled OR, which was then used as the measure for the overall population of the study. The I^2^ statistic was used to test for the heterogeneity across studies. If no evidence of heterogeneity was presented (I^2^ < 50%), a fixed effect model was used. Otherwise, a random effect model was adopted. Sensitivity analysis was conducted by excluding one study at a time to determine whether the results of this meta-analysis were robust. Subgroup analyses based on study design and age groups of participants were further performed. Publication bias was assessed when there were no fewer than 10 studies, via Begg’s rank correlation and Egger’s linear regression methods. Probability value P < 0.05 was considered statistically significant. All statistical analyses were performed using STATA software (version 14.0; Stata Corporation, College Station, Texas, USA).

## Results

### Literature selection

**Fig. 1:**
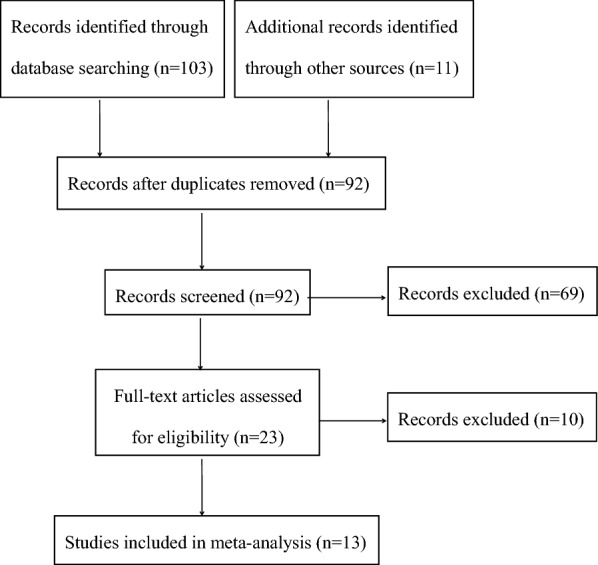
Flow diagram of literature selection

A total of 114 studies were identified, of which 103 studies were identified through database searching, 11 studies were identified by reference lists checking. After removal of duplicates, the titles and abstracts of the remaining 92 studies were screened for eligibility. The full texts of the 23 potentially related studies were then strictly assessed. Finally, 13 studies were included in the meta-analysis (Fig. 1).

### Study characteristics and quality evaluation

All 13 studies included in the final meta-analysis were published in English. Of these, 2 [23, 32] were case–control studies, 6 [12, 28, 29, 33–35] were cohort studies, 5 [10, 11, 25–27] were cross-sectional studies. A total of 100,137 subjects were involved in this meta-analysis, ranging in age from 5 to 75 years. All included studies adopted self-reported asthma except for 3 studies [23, 29, 32]. Among the anthropometric measures of abdominal obesity, WC was the most commonly used one. All 13 studies adopted WC as the measure or one of the measures of abdominal obesity, while only 3 studies [23, 32, 34] also used WHtR, 2 studies [25, 34] also used WHR, 1 study [32] also used CI. When reporting the final results, 6 studies [11, 23, 32–35] reported the results for overall population, 4 studies [12, 25–27] reported the results for males and females separately, 3 studies [10, 28, 29] reported both the results for overall population and the results for males and females. The main characteristics of the included studies are showed in Table 1.

**Table 1:** The main characteristics of the included studies

First author (year)	Region	Study design	Sample size	Participants	Anthropometric measures of abdominal obesity	Results	
Papoutsakis (2015), [23]	Greece	Case–control	514	5–11 years old	WC WHtR	WC	1.99 (1.07, 3.68)
						WHtR	2.24 (1.47, 3.40)
Musaad (2009), [32]	US	Case–control	584	5–18 years old	WC WHtR CI	WC	1.20 (0.49, 2.92)
						WHtR	2.00 (0.93, 4.31)
						CI	2.63 (1.19, 5.82)
Leone (2012), [33]	French	Cohort	6267	≥ 65 years old	WC	WC	3.84 (1.55, 9.49)
Egan (2015), [28]	Norway	Cohort	3139	12–30 years old	WC	WC	1.38 (1.07, 1.78)
						WC (male)	1.80 (1.20, 2.71)
						WC (female)	1.11 (0.80, 1.54)
Chen (2014), [34]	China	Cohort	2758	4th to 6th grade	WC WHR WHtR	WC	1.22 (0.60, 2.50)
						WHtR	1.17 (0.57, 2.39)
						WHR	1.53 (0.64, 3.66)
Brumpton (2013), [35]	Norway	Cohort	23,191	19–55 years old	WC	WC	1.62 (1.36, 1.94)
Brumpton (2013), [35]	Norway	Cohort	23,245	19-65 years old	WC	WC (male)	0.88 (0.53, 1.47)
						WC (female)	1.46 (1.04, 2.05)
Kronander (2004), [29]	Sweden	Cohort	4178	20–50 years old	WC	WC	2.60 (1.32, 5.12)
						WC (male)	5.21 (1.52, 17.84)
						WC (female)	1.80 (0.78, 4.12)
Turley (2006), [26]	New Zealand	Cross-sectional	10,026	25–44 years old	WC	WC (male)	1.60 (0.90, 2.30)
						WC (female)	1.40 (1.00, 1.80)
Appleton (2006), [25]	Australia	Cross-sectional	4060	≥ 18 years old	WC WHR	WC (male)	1.10 (0.70, 1.70)
						WC (female)	1.50 (1.00, 2.10)
						WHR (male)	1.30 (0.70, 2.50)
						WHR (female)	1.50 (1.00, 2.20)
Egan (2014), [10]	Norway	Cross-sectional	15,625	12–19 years old	WC	WC	1.36 (1.16, 1.60)
						WC (male)	1.38 (1.12, 1.70)
						WC (female)	1.33 (1.04, 1.69)
Ma (2010), [11]	US	Cross-sectional	4493	≥ 20 years old	WC	WC	1.75 (1.22, 2.51)
Chen (2005), [27]	Canada	Cross-sectional	2057	18–79 years old	WC	WC (male)	0.99 (0.51, 1.93)
						WC (female)	1.87 (1.18, 2.98)

The quality of the 6 cohort studies and the 2 case–control studies were assessed by the NOS. Three studies [23, 32, 34] got a score of 6, three studies [28, 29, 33] got a score of 7, two studies [12, 35] got a score of 9, indicating that both the cohort studies and the case control studies owned fairly high quality. For the 5 cross-sectional studies, two items (item 4 and 11) in the AHRQ recommended criteria were not suitable. For item 1, 2, 8, as well as item 10, all answers were “yes”. For item 3, all answers were “yes” except for one study [25]. For item 5, all answers were “no”. The detailed assessment of the 5 cross-sectional studies are presented in Table 2.

**Table 2:** Quality assessment of the 5 cross-sectional studies according to AHRQ recommended criteria

Studies	1	2	3	4	5	6	7	8	9	10	11
Turley et al. [26]	√	√	√	–	×	√	√	√	×	√	–
Appleton et al. [25]	√	√	?	–	×	√	?	√	×	√	–
Egan et al. [28]	√	√	√	–	×	×	?	√	?	√	–
Ma et al. [11]	√	√	√	–	×	√	√	√	?	√	–
Chen et al. [27]	√	√	√	–	×	×	?	√	?	√	–

1–11 represents the 11 items of the AHRQ recommended criteria

“√” means “yes”, “×” means “no”, “?” means “unclear”, “–” means “not suitable”

### Association between abdominal obesity and asthma

**Fig. 2:**
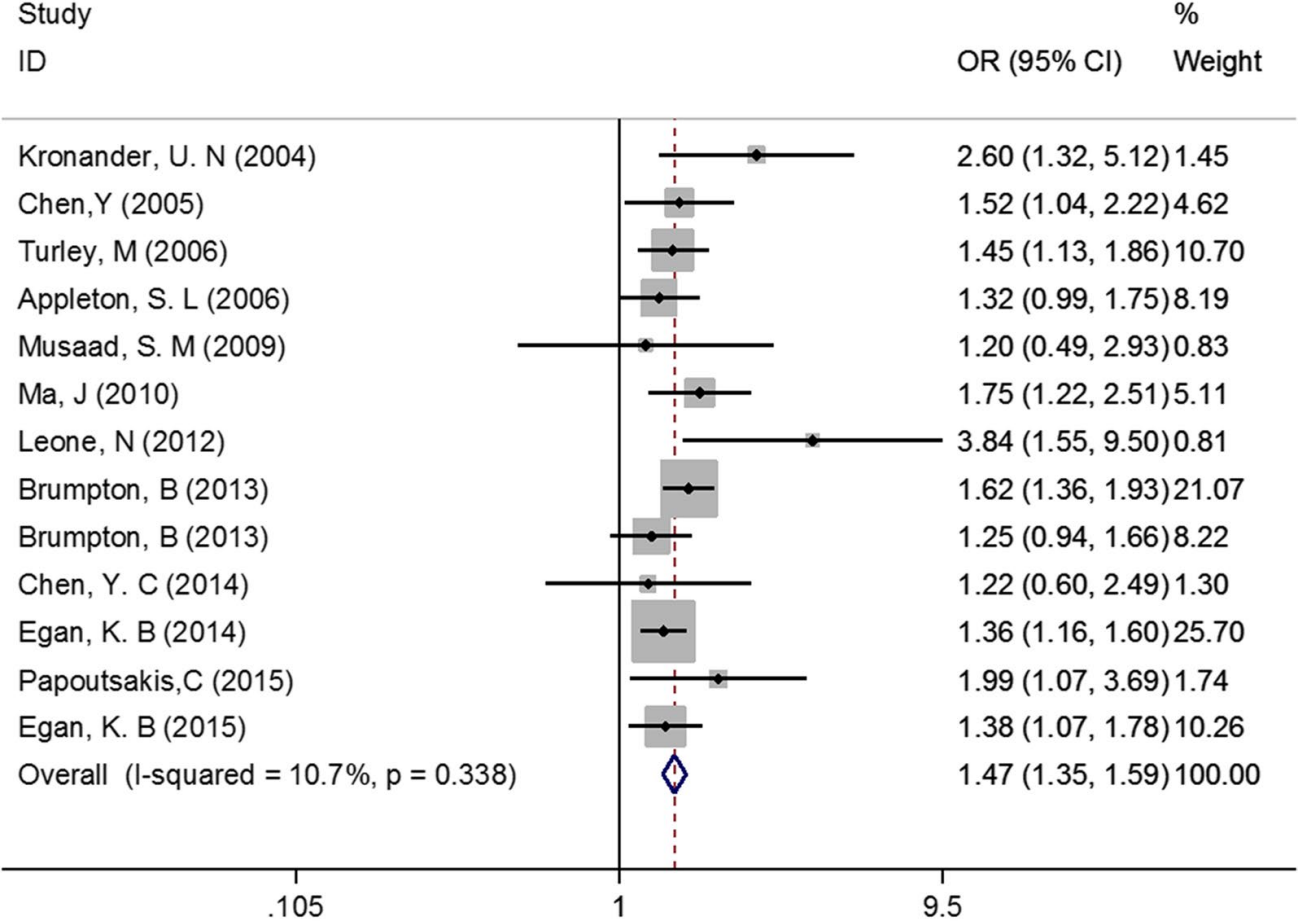
Association between abdominal obesity and asthma

Only data of WC were analyzed in this meta-analysis, since measures other than WC were rarely used in the 13 included studies. Our meta-analysis observed a positive association between abdominal obesity and asthma (P < 0.001, OR = 1.47, 95% CI 1.35–1.59; Fig. 2). No evidence of heterogeneity (I^2^ = 10.7%) or publication bias (Begg’s test P = 0.200, Egger’s test P = 0.146) was found. After sequentially excluding each included study in sensitivity analysis, the results remained largely unchanged, indicating that the meta-analysis was stable.

**Fig. 3:**
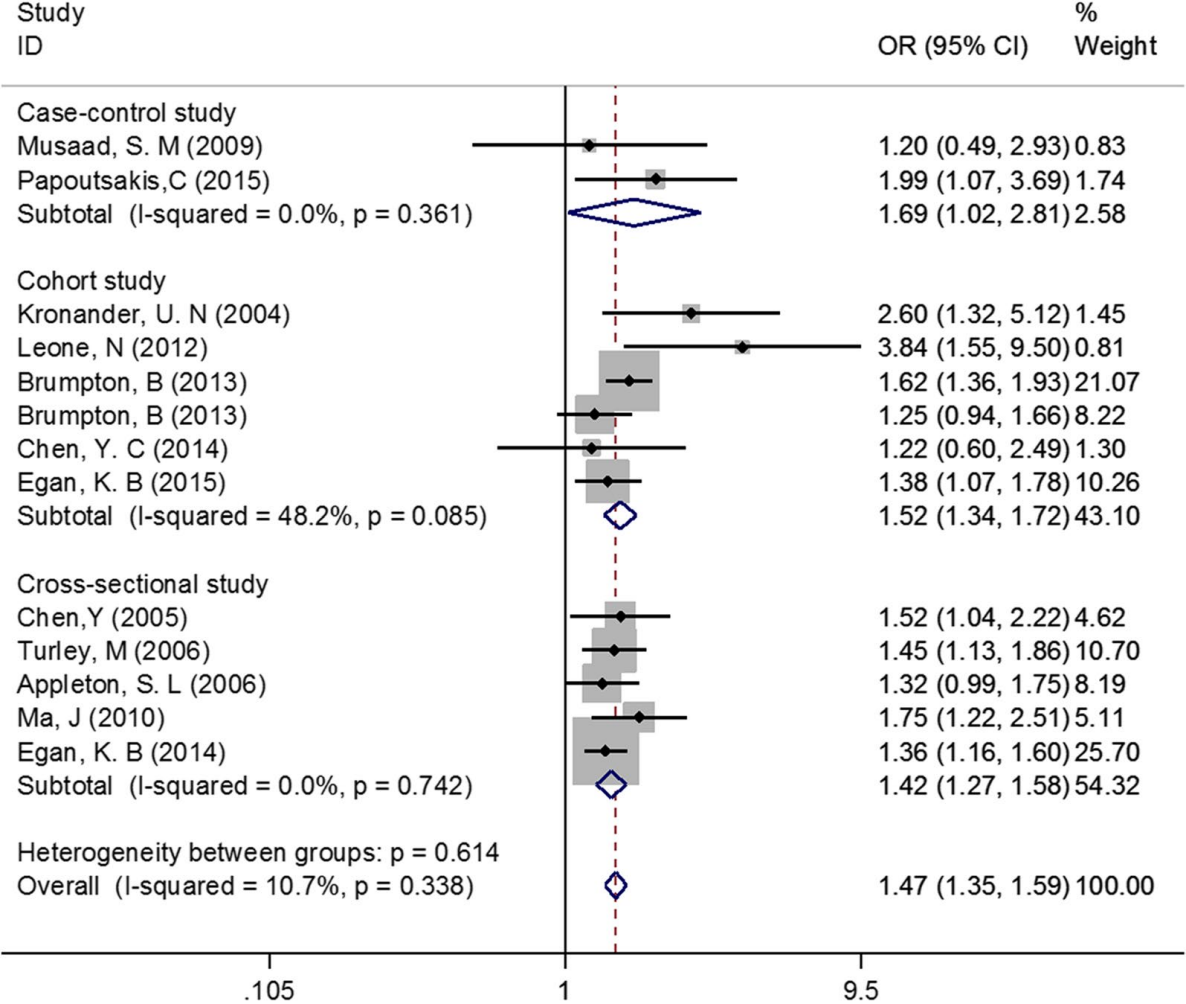
Subgroup analysis by study design

**Fig. 4:**
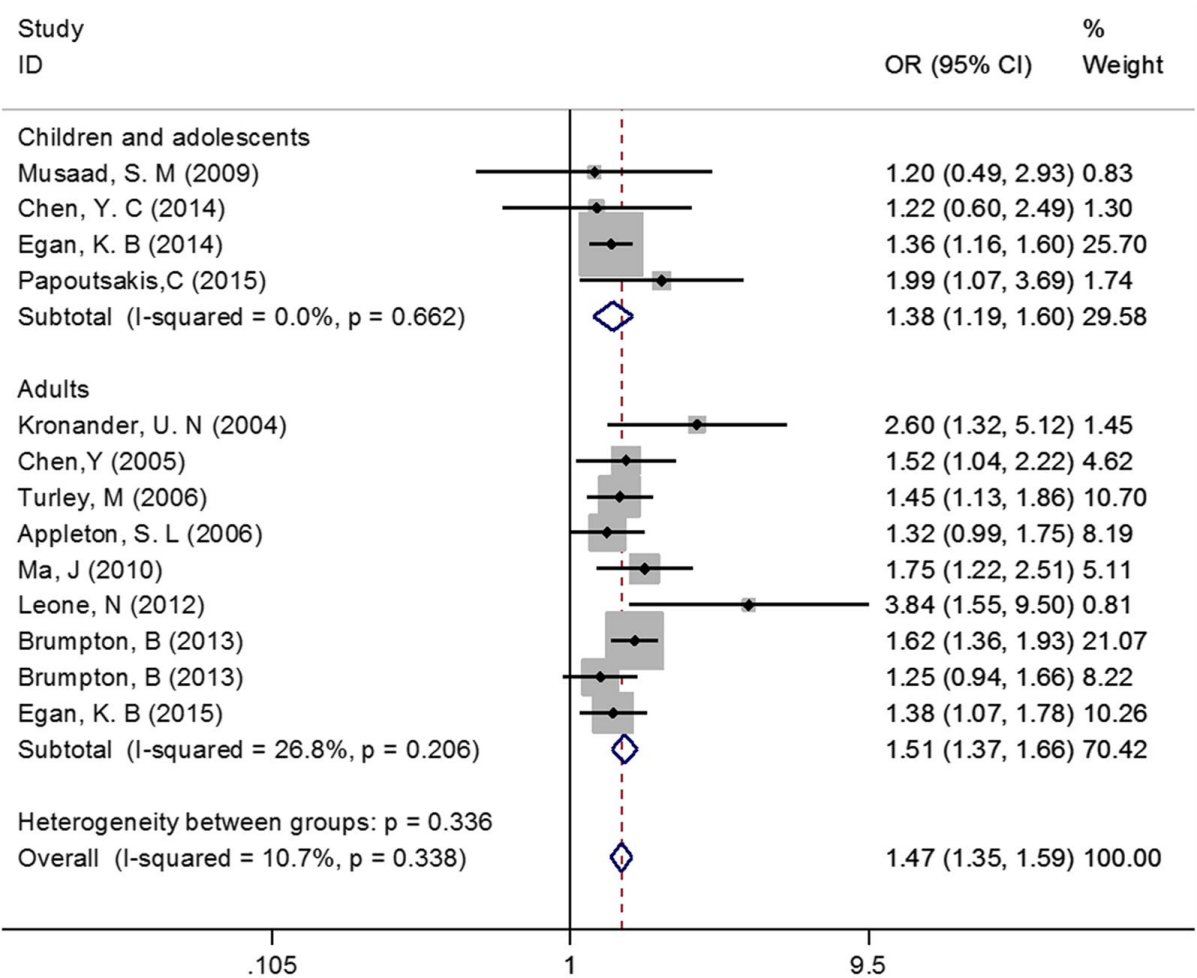
Subgroup analysis by age groups of participants

Considering that the 13 studies had different types of study design and focused on different age groups of participants, we conducted subgroup analyses to further evaluate the association between abdominal obesity and asthma. Subgroup analysis by study design obtained consistently positive results among the three subgroups (Case–control study: OR = 1.69, 95% CI 1.02–2.81, Cohort study: OR = 1.52, 95% CI 1.34–1.72, Cross-sectional study: OR = 1.42, 95% CI 1.27–1.58, Fig. 3). Similarly, subgroup analysis by age groups of participants also observed consistent results across subgroups (Children and adolescents: OR = 1.38, 95% CI 1.19–1.60, Adults: OR = 1.51, 95% CI 1.37–1.66, Fig. 4).

It is noted that 2 [12, 33] of the 13 included studies reported the association between abdominal obesity and asthma after additional adjustment for BMI. However, meta-analysis was not pursued owing to the small number of studies and the considerable heterogeneity (I^2^ = 81.4%). Of the 2 studies, one study [33] was conducted based on a large elderly cohort, showing that abdominal obesity was independently and strongly related to incident asthma (OR = 3.84, 95% CI 1.55–9.49). Another study [12] reported results separately for adult males and females, showing that abdominal obesity remained a risk factor for incident asthma in females after adjusting the effect of BMI (Females: OR = 1.46, 95% CI 1.04–2.05; Males: OR = 0.88, 95% CI 0.53–1.47).

### Sex-specific association between abdominal obesity and asthma

A total of 7 studies reported data separately for males and females. Therefore, the sex-specific association between abdominal obesity and asthma was further explored in this meta-analysis.

**Fig. 5:**
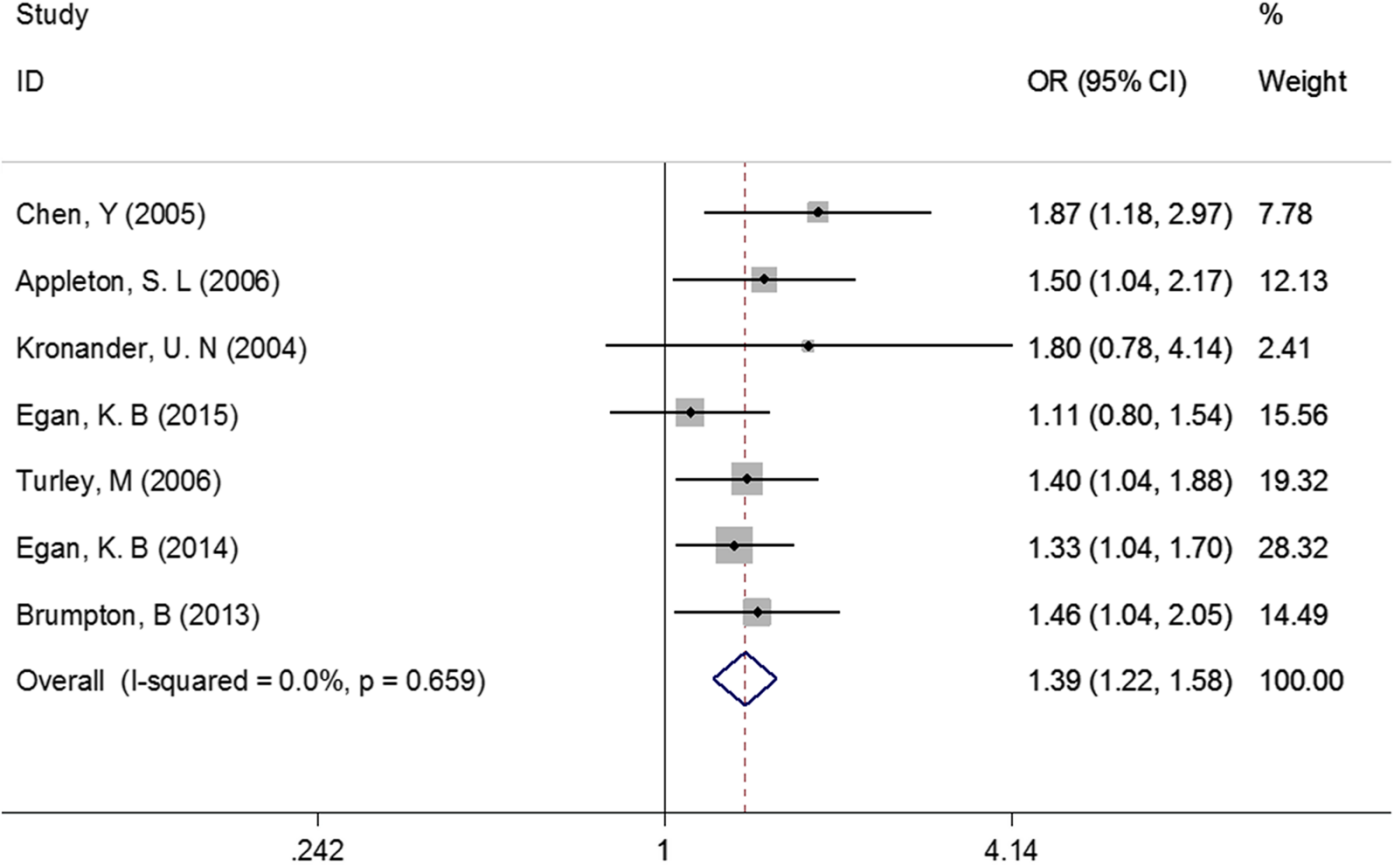
Association between abdominal obesity and asthma in females

**Fig. 6:**
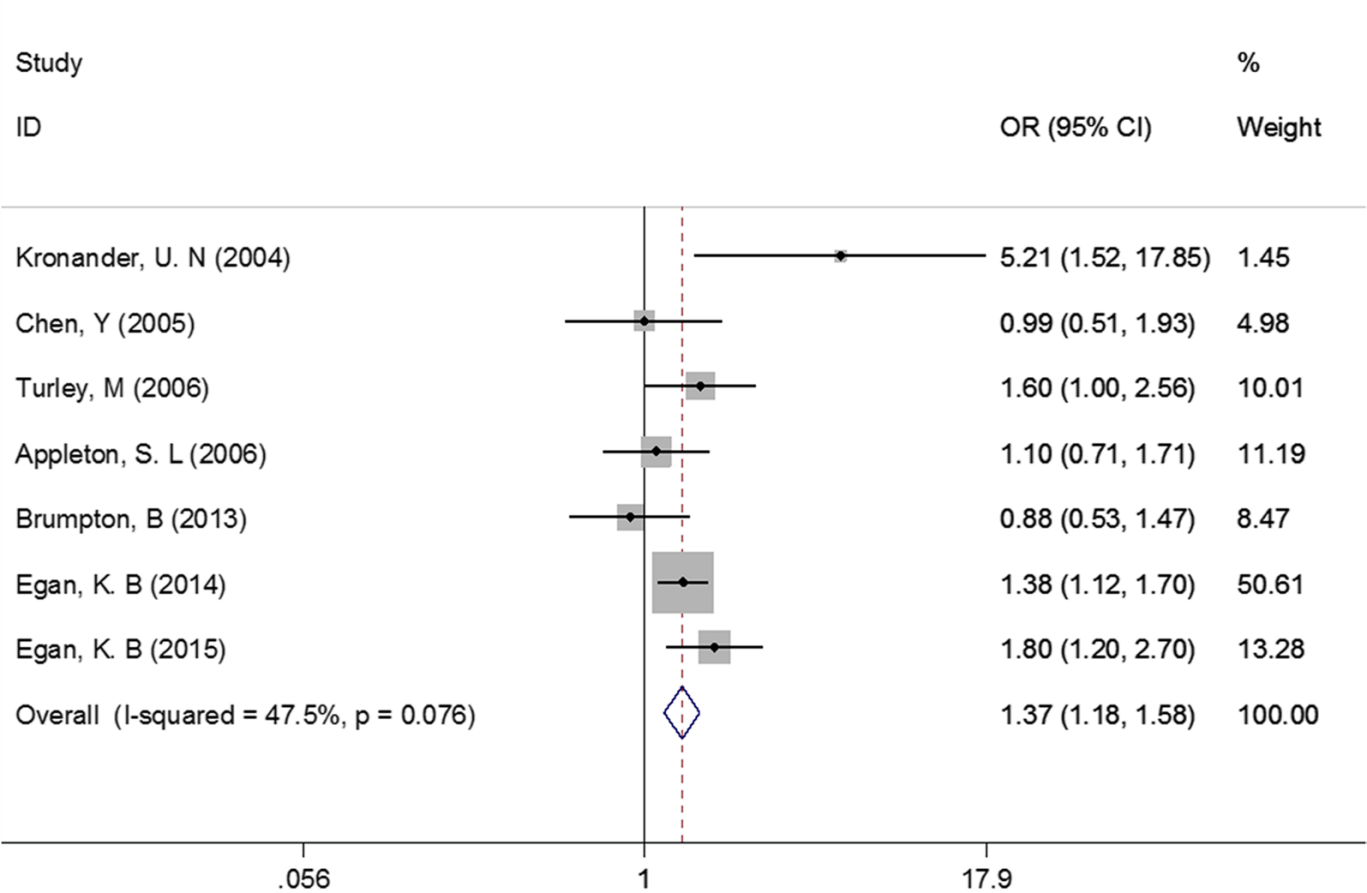
Association between abdominal obesity and asthma in males

For females, as showed in Fig. 5, the pooled OR and 95% CI was 1.39 (1.22, 1.58), P < 0.001. No evidence of heterogeneity was observed in this meta-analysis (I^2^ = 0.0%). No significant change was found in sensitivity analysis. For males, similar results were observed (OR = 1.37, 95% CI 1.18–1.58, P < 0.001; Fig. 6). No significant change was found in sensitivity analysis, either.

### Association between abdominal overweight and asthma

**Fig. 7:**
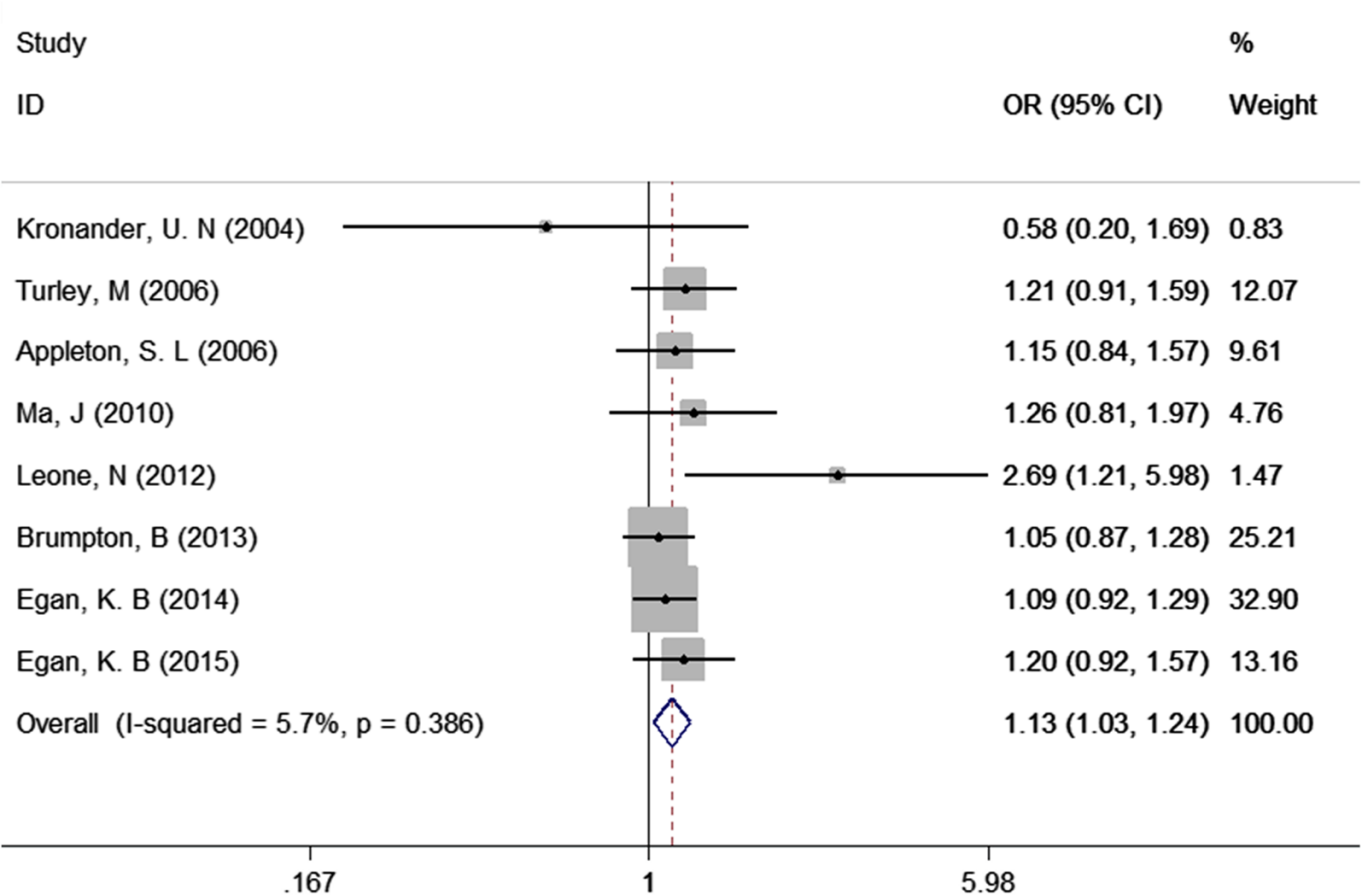
Association between abdominal overweight and asthma

Notably, of the 13 included studies, 8 studies [10–12, 25, 26, 28, 29, 33] also reported the association between abdominal overweight and asthma. Therefore, this association was further explored in our meta-analysis. The results of our meta-analysis showed that the association between abdominal overweight and asthma (OR = 1.13, 95% CI 1.03–1.24, P = 0.014, Fig. 7) was weaker than the association between abdominal obesity and asthma, indicating that there is a dose–response effect in the relationship between abdominal weight status and asthma.

In addition, since the search strategy of our meta-analysis was not targeted at abdominal overweight, we did a further literature search on abdominal overweight and asthma to determine whether there were any missing studies in the above meta-analysis. Finally, no additional eligible study was found, which suggests that our results are credible.

## Discussion

Unlike previous meta-analyses published in this field, which focused on the association between general obesity (measured by BMI) and asthma [14–17], our meta-analysis focused on the association between abdominal obesity (measured by WC) and asthma. The findings of our meta-analysis show that there is a positive association between abdominal obesity and asthma (OR = 1.47, 95% CI 1.35–1.59). Moreover, this association is similar in males (OR = 1.37, 95% CI 1.18–1.58) and females (OR = 1.39, 95% CI 1.22–1.58). Although inadequately reported, the association between abdominal obesity and asthma may be independent of BMI. In addition, this meta-analysis also suggests that there is a dose–response relationship between abdominal weight status and asthma.

Therefore, on the basis of our findings, addressing abdominal obesity issue is of great importance. There is growing evidence showing that in addition to the increased risk of developing asthma, abdominal obesity is also associated with lower lung function, more severe asthma symptoms, as well as more poorly controlled asthma [13, 32, 36, 37]. Notably, studies on weight loss interventions among obese asthmatic patients consistently showed a significant improvement in asthma symptoms as well as asthma control after weight loss [38–41]. Therefore, in the light of these evidence, efforts should be made to normalize body fat distribution among people who are abdominally obese, in order to reduce the risk of asthma as well as to improve the prognosis of asthma.

Although the clear mechanism underlying the association between abdominal obesity and asthma remains largely unknown, hypotheses can be suggested. On the one hand, the accumulation of abdominal adipose tissue mechanically restricts the diaphragm and limits lung expansion, resulting in reduced functional residual capacity and tidal volume, which further leads to the conversion of airway smooth muscle from rapidly cycling actin-myosin cross bridges to slowly cycling latch bridges [42–44]. The resultant latch state may further lead to persistent airway obstruction and increased airway responsiveness [42, 45]. On the other hand, adipose tissue, particularly visceral adipose tissue, is closely related to the secretion of pro-inflammatory mediators, such as leptin, adiponectin, interleukin-6, and tumor necrosis factor α, which may directly affect the airway or bring about changes in immune responses [19, 33, 35, 46]. Moreover, considerable overlap in the genetic determinants of asthma and obesity has been observed in genetic epidemiological studies [42, 45, 47, 48]. For example, genes encoding for the β_2_ adrenergic receptor and tumour necrosis factor ɑ have been found to be strongly associated with both obesity and asthma [42, 45]. Some obesity candidate genes, such as genes encoding for the glucocorticoid receptor and insulin-like growth factor 1, have been found to be clustered in chromosomal regions associated with asthma [42, 45]. Besides, some obesity candidate genes can encode protein products that may directly affect asthma, such as the cytokines mentioned above [44]. In addition, some comorbidities of obesity, such as insulin-resistance, gastroesophageal reflux disease, and obstructive sleep apnea, may also have an effect on asthma [33, 44].

There are some limitations in this meta-analysis. First, due to the lack of study on the association between abdominal obesity and asthma, we included all available relevant original epidemiological studies (of any design) in this meta-analysis. As a result, three types of study, cross-sectional study, cohort study, as well as case–control study, were included in our meta-analysis. However, different types of study may reflect different indexes of asthma. For example, of the studies included in our meta-analysis, 5 cross-sectional studies reflected the association between abdominal obesity and the current prevalence of asthma, while the 6 cohort studies reported the association between abdominal obesity and the incidence of asthma. Therefore, the results of our meta-analysis cannot be simply interpreted as abdominal obesity is associated with the prevalence or with the incidence of asthma. It may be more appropriate to consider our results as the association between abdominal obesity and the overall risk of asthma. However, according to our subgroup analysis by study design, all three subgroups obtained statistically significant results, indicating that abdominal obesity may be associated with both the prevalence and the incidence of asthma. More studies are needed in the future to clarify the association between abdominal obesity and asthma.

Second, although our meta-analysis focused on the association between abdominal obesity (measured by WC) and asthma, the results of our meta-analysis were similar to those of previous meta-analyses that focused on general obesity (measured by BMI). Therefore, whether abdominal obesity add further information to the currently recognized relationship between general obesity and asthma is in doubt. However, based on the current evidence, we cannot resolve this doubt in this meta-analysis. But among the 13 included studies, 2 studies [12, 33] reported the results after additional adjustment for BMI. They found that the association between abdominal obesity and asthma remained significant after adjustment for BMI, indicating that abdominal obesity may be independently associated with asthma. The small number of studies and the considerable heterogeneity precluded further meta-analysis. More studies are needed in the future to determine whether the association between abdominal obesity and asthma is independent of BMI.

Third, in the majority of the included studies (10 of 13 studies), the definition of asthma was based on self-report, which was subject to reporting bias and misclassification. However, self-reported asthma is widely used in epidemiological studies and has been evaluated as a reliable and valid measure for asthma [49–51]. Moreover, to reduce the potential risk of bias and misclassification caused by self-reported asthma, several studies [11, 12, 33, 35] further conducted sensitivity analysis using a more stringent definition of asthma and found that the results were almost unchanged.

Fourth, only data of WC were adopted in our meta-analysis as there were few studies have provided data on other abdominal obesity measures. However, using multiple measures of abdominal obesity to comprehensively evaluate the association between abdominal obesity and asthma is also desirable. Therefore, future studies can use other measures of abdominal obesity to better explore this relationship.

Despite the limitations mentioned above, there are also some strengths in this meta-analysis. First, different from previous meta-analyses that focused on the association between general obesity (measured by BMI) and asthma [14–17], our meta-analysis focused on the association between abdominal obesity (measured by WC) and asthma. Sex-specific association was further explored in this meta-analysis. Second, the association between abdominal overweight and asthma was also explored. A dose–response relationship between abdominal weight status and asthma was further observed in this meta-analysis. Third, subgroup analyses based on study design and age groups of participants were further conducted and consistently positive results across subgroups were observed, which provided further support for the positive association between abdominal obesity and asthma. Fourth, all 13 studies included in our meta-analysis owned fairly high quality. Fifth, most of the studies included in this meta-analysis had large sample sizes, which may allow a much greater possibility of drawing correct conclusions. Finally, all included studies measured WC objectively by trained researchers, which avoided the impact of reporting bias.

## Conclusions

This meta-analysis shows a positive association between abdominal obesity and asthma. Moreover, this association is similar in males and females. In addition, this meta-analysis suggests that there is a dose–response relationship between abdominal weight status and asthma. Therefore, addressing abdominal obesity issue is of great importance. More studies are needed in the future to clarify the association between abdominal obesity and asthma. Studies adopting other measures of abdominal obesity are also desirable.

## Abbreviations

OR: odds ratio
RR: risk ratio
CI: confidence interval
BMI: body mass index
WC: waist circumference
WHtR: waist to height ratio
WHR: waist hip ratio
CI: conicity index
NOS: Newcastle–Ottawa Scale
AHRQ: Agency for Healthcare Research and Quality

## References

[1] Soriano JB, Abajobir AA, Abate KH, Abera SF, Agrawal A, Ahmed MB (2017). Global, regional, and national deaths, prevalence, disability-adjusted life years, and years lived with disability for chronic obstructive pulmonary disease and asthma, 1990–2015: a systematic analysis for the Global Burden of Disease Study 2015. Lancet Respir Med.

[2] Masoli M, Fabian D, Holt S, Beasley R (2004). The global burden of asthma: executive summary of the GINA Dissemination Committee report. Allergy.

[3] To T, Stanojevic S, Moores G, Gershon AS, Bateman ED, Cruz AA (2012). Global asthma prevalence in adults: findings from the cross-sectional world health survey. BMC Public Health.

[4] Vos T, Allen C, Arora M, Barber RM, Bhutta ZA, Brown A (2016). Global, regional, and national incidence, prevalence, and years lived with disability for 310 diseases and injuries, 1990–2015: a systematic analysis for the Global Burden of Disease Study 2015. Lancet.

[5] Accordini S, Corsico AG, Braggion M, Gerbase MW, Gislason D, Gulsvik A (2013). The cost of persistent asthma in Europe: an international population-based study in adults. Int Arch Allergy Immunol.

[6] Nurmagambetov T, Kuwahara R, Garbe P (2018). The economic burden of asthma in the United States, 2008–2013. Ann Am Thorac Soc.

[7] Afshin A, Forouzanfar MH, Reitsma MB, Sur P, Estep K, Lee A (2017). Health effects of overweight and obesity in 195 countries over 25 years. N Engl J Med.

[8] Kyrgiou M, Kalliala I, Markozannes G, Gunter MJ, Paraskevaidis E, Gabra H (2017). Adiposity and cancer at major anatomical sites: umbrella review of the literature. BMJ.

[9] Dixon JB (2010). The effect of obesity on health outcomes. Mol Cell Endocrinol.

[10] Egan KB, Ettinger AS, DeWan AT, Holford TR, Holmen TL, Bracken MB (2014). General, but not abdominal, overweight increases odds of asthma among Norwegian adolescents: the Young-HUNT study. Acta Paediatr.

[11] Ma J, Xiao L, Knowles SB (2010). Obesity, insulin resistance and the prevalence of atopy and asthma in US adults. Allergy.

[12] Brumpton B, Langhammer A, Romundstad P, Chen Y, Mai XM (2013). General and abdominal obesity and incident asthma in adults: the HUNT study. Eur Respir J.

[13] Von Behren J, Lipsett M, Horn-Ross PL, Delfino RJ, Gilliland F, McConnell R (2009). Obesity, waist size and prevalence of current asthma in the California Teachers Study cohort. Thorax.

[14] Beuther DA, Sutherland ER (2007). Overweight, obesity, and incident asthma. Am J Resp Crit Care.

[15] Flaherman V (2006). A meta-analysis of the effect of high weight on asthma. Arch Dis Child.

[16] Azizpour Y, Delpisheh A, Montazeri Z, Sayehmiri K, Darabi B (2018). Effect of childhood BMI on asthma: a systematic review and meta-analysis of case-control studies. BMC Pediatr.

[17] Chen YC, Dong GH, Lin KC, Lee YL (2013). Gender difference of childhood overweight and obesity in predicting the risk of incident asthma: a systematic review and meta-analysis. Obes Rev.

[18] Daniels SR, Khoury PR, Morrison JA (1997). The utility of body mass index as a measure of body fatness in children and adolescents: differences by race and gender. Pediatrics.

[19] Tchernof A, Després J (2013). Pathophysiology of human visceral obesity: an update. Physiol Rev.

[20] Ashwell M, Gunn P, Gibson S (2012). Waist-to-height ratio is a better screening tool than waist circumference and BMI for adult cardiometabolic risk factors: systematic review and meta-analysis. Obes Rev.

[21] Yusuf S, Hawken S, Ounpuu S, Bautista L, Franzosi MG, Commerford P (2005). Obesity and the risk of myocardial infarction in 27,000 participants from 52 countries: a case-control study. Lancet.

[22] Wang Y, Rimm EB, Stampfer MJ, Willett WC, Hu FB (2005). Comparison of abdominal adiposity and overall obesity in predicting risk of type 2 diabetes among men. Am J Clin Nutr.

[23] Papoutsakis C, Chondronikola M, Antonogeorgos G, Papadakou E, Matziou V, Drakouli M (2015). Associations between central obesity and asthma in children and adolescents: a case-control study. J Asthma.

[24] Benedetti FJ, Bosa VL, Mariante Giesta J, Fischer GB (2015). Anthropometric indicators of general and central obesity in the prediction of asthma in adolescents, Central obesity in asthma. Nutr Hosp.

[25] Appleton SL, Adams RJ, Wilson DH, Taylor AW, Ruffin RE (2006). Central obesity is associated with nonatopic but not atopic asthma in a representative population sample. J Allergy Clin Immunol.

[26] Turley M, Tobias M, Paul S (2006). Non-fatal disease burden associated with excess body mass index and waist circumference in New Zealand adults. Aust N Z J Public Health.

[27] Chen Y, Rennie D, Cormier Y, Dosman J (2005). Sex specificity of asthma associated with objectively measured body mass index and waist circumference—the Humboldt study. Chest.

[28] Egan KB, Ettinger AS, DeWan AT, Holford TR, Holmen TL, Bracken MB (2015). Longitudinal associations between asthma and general and abdominal weight status among Norwegian adolescents and young adults: the HUNT Study. Pediatr Obes.

[29] Kronander UN, Falkenberg M, Zetterstrom O (2004). Prevalence and incidence of asthma related to waist circumference and BMI in a Swedish community sample. Resp Med.

[30] Wells GA, Shea B, O’Connell D, Peterson J, Welch V, Losos M, et al. NOS Manual. In: The Newcastle-Ottawa Scale (NOS) for assessing the quality of nonrandomized studies in meta-analyses. The Ottawa Hospital Research Institute. 2018. http://www.ohri.ca/programs/clinical_epidemiology/oxford.asp. Accessed 13 Mar 2018.

[31] Rostom A, Dubé C, Cranney A, Saloojee N, Sy R, Garritty C, et al. Appendix D. Quality assessment forms. In: Celiac disease. Agency for Healthcare Research and Quality (US). 2004. https://www.ncbi.nlm.nih.gov/books/NBK35156/. Accessed 13 Mar 2018.

[32] Musaad SMA, Patterson T, Ericksen M, Lindsey M, Dietrich K, Succop P (2009). Comparison of anthropometric measures of obesity in childhood allergic asthma: central obesity is most relevant. J Allergy Clin Immunol.

[33] Leone N, Courbon D, Berr C, Barberger-Gateau P, Tzourio C, Alperovitch A (2012). Abdominal obesity and late-onset asthma: cross-sectional and longitudinal results: the 3C study. Obesity..

[34] Chen YC, Tu YK, Huang KC, Chen PC, Chu DC, Lee YL (2014). Pathway from central obesity to childhood asthma. Physical fitness and sedentary time are leading factors. Am J Respir Crit Care Med.

[35] Brumpton BM, Camargo CJ, Romundstad PR, Langhammer A, Chen Y, Mai XM (2013). Metabolic syndrome and incidence of asthma in adults: the HUNT study. Eur Respir J.

[36] Capelo AV, de Fonseca VM, Peixoto MV, de Carvalho SR, Guerino LG (2015). Central obesity and other factors associated with uncontrolled asthma in women. Allergy Asthma Clin Immunol.

[37] Vatrella A, Calabrese C, Mattiello A, Panico C, Costigliola A, Chiodini P (2016). Abdominal adiposity is an early marker of pulmonary function impairment: findings from a Mediterranean Italian female cohort. Nutr Metab Cardiovasc Dis.

[38] Van Leeuwen JC, Hoogstrate M, Duiverman EJ, Thio BJ (2014). Effects of dietary induced weight loss on exercise-induced bronchoconstriction in overweight and obese children. Pediatr Pulmonol.

[39] Dias-Junior SA, Reis M, de Carvalho-Pinto RM, Stelmach R, Halpern A, Cukier A (2014). Effects of weight loss on asthma control in obese patients with severe asthma. Eur Respir J.

[40] Jensen ME, Gibson PG, Collins CE, Hilton JM, Wood LG (2013). Diet-induced weight loss in obese children with asthma: a randomized controlled trial. Clin Exp Allergy.

[41] Juel CT, Ali Z, Nilas L, Ulrik CS (2012). Asthma and obesity: does weight loss improve asthma control? a systematic review. J Asthma Allergy.

[42] Beuther DA, Weiss ST, Sutherland ER (2006). Obesity and asthma. Am J Respir Crit Care Med.

[43] Fredberg JJ, Inouye D, Miller B, Nathan M, Jafari S, Raboudi SH (1997). Airway smooth muscle, tidal stretches, and dynamically determined contractile states. Am J Respir Crit Care Med.

[44] Shore SA (2008). Obesity and asthma: possible mechanisms. J Allergy Clin Immunol.

[45] Tantisira KG, Weiss ST (2001). Complex interactions in complex traits: obesity and asthma. Thorax.

[46] Lessard A, Almeras N, Turcotte H, Tremblay A, Despres JP, Boulet LP (2011). Adiposity and pulmonary function: relationship with body fat distribution and systemic inflammation. Clin Invest Med.

[47] Hallstrand TS, Fischer ME, Wurfel MM, Afari N, Buchwald D, Goldberg J (2005). Genetic pleiotropy between asthma and obesity in a community-based sample of twins. J Allergy Clin Immunol.

[48] Szczepankiewicz A, Bręborowicz A, Sobkowiak P, Popiel A (2009). Are genes associated with energy metabolism important in asthma and BMI?. J Asthma.

[49] Toren K, Brisman J, Jarvholm B (1993). Asthma and asthma-like symptoms in adults assessed by questionnaires. Chest.

[50] De Marco R, Cerveri I, Bugiani M, Ferrari M, Verlato G (1998). An undetected burden of asthma in Italy: the relationship between clinical and epidemiological diagnosis of asthma. Eur Respir J.

[51] Braun-Fahrlander C, Gassner M, Grize L, Minder CE, Varonier HS, Vuille JC (1998). Comparison of responses to an asthma symptom questionnaire (ISAAC core questions) completed by adolescents and their parents. Pediatr Pulmonol.

